# Autologous porcine VRAM flap model for VCA research

**DOI:** 10.3389/frtra.2024.1504959

**Published:** 2024-12-06

**Authors:** Caitlin M. Blades, Zari P. Dumanian, Yong Wang, Zhaohui Wang, Bing Li, Kia M. Washington, Julia B. Slade, Conor L. Evans, Paula Arrowsmith, Evan A. Farkash, Jason W. Yu, Mark A. Greyson, Christene A. Huang, Nalu Navarro-Alvarez, David W. Mathes

**Affiliations:** ^1^ Department of Surgery, University of Colorado Denver Anschutz Medical Campus, Aurora, CO, United States; ^2^ Wellman Center for Photomedicine, Massachusetts General Hospital, Harvard Medical School, Charlestown, MA, United States; ^3^ Department of Pathology, University of Michigan School of Medicine, Ann Arbor, MI, United States

**Keywords:** vertical rectus abdominis myocutaneous flap, autologous, swine, ischemia, neck flap

## Abstract

**Introduction:**

As research advances in vascularized composite allotransplantation (VCA), large animal models are essential for translational studies related to immune rejection and graft survival. However, procurement of large flaps can cause significant defects, complicating wound closure and increasing postoperative risks. This study details the surgical techniques and outcomes of autologous vertical rectus abdominis myocutaneous (VRAM) flap transplantation and neck flap isolation with induced ischemia in a swine model. The purpose of this study was to identify the most effective control procedure for use in future VRAM flap allotransplantation research.

**Methods:**

We performed two left heterotopic autologous VRAM flap transplants and two right anterolateral neck flap isolations using female Yucatan pigs. Postoperatively, animals were monitored for complications and flap healing, with punch biopsies taken on POD1, 5, and at the end of the study for histological analysis. Transcutaneous oxygen and temperature were also recorded.

**Results:**

Both autologous flaps survived after vessel anastomosis, with effective closure of abdominal defects using suturable mesh, and no postoperative complications were observed. Histology revealed mild dermal edema and perivascular inflammation on POD5. In the neck flap group, both flaps survived temporary ischemia, however, postoperative complications included dorsal flap necrosis and wound dehiscence, requiring reoperation. No gross inflammation or edema was observed following surgery and histologically there was only mild dermal edema on POD5.

**Discussion:**

We have developed a low-risk, technically feasible porcine autologous VRAM flap transplantation model and our findings support its use in future VCA studies.

## Introduction

The vertical rectus abdominis myocutaneous (VRAM) flap offers a reliable blood supply and substantial bulk, making it a popular choice for reconstructing tissue defects in the breast, chest wall, groin, hip, and perineum (1). For these reasons, it is frequently used for reconstruction following advanced breast cancer (2). As a Mathes and Nahai type III flap, it receives dual perfusion from the deep superior and deep inferior epigastric arteries (3). This robust vascular supply makes the VRAM flap highly versatile; it can be transferred superiorly or inferiorly and used as a free flap (4). Unfortunately, not all patients requiring reconstruction have adequate autogenous tissue. Thus, exploring alternative treatment options, such as vascularized composite allotransplantation (VCA), is essential for these individuals.

VCA involves the transplantation of multi-layered, complex tissues, such as hands, face, and penis, to restore structure and functionality in patients with significant tissue loss (5, 6). Compared to solid organ transplantation, VCA is a relatively new field that has not been as thoroughly researched, leaving many questions unanswered. Current VCA studies mainly focus on developing protocols to prolong graft survival and reduce or eliminate the need for immunosuppression (7–9). These studies often use large animal models due to their anatomical and physiological similarities to humans (10). Several clinically relevant animal VCA models have been established (11–13), and their success is frequently evaluated in comparison to outcomes from autologous control groups.

The significance of incorporating a surgical control group in VCA animal studies cannot be overstated. It enables researchers to accurately identify and establish baseline measurements for postoperative inflammation and tissue injury directly attributable to the surgical intervention itself. Given that the immune response elicited by surgery can significantly contribute to postoperative morbidity and mortality (14), it is crucial to distinguish this response from that associated with immune rejection. Furthermore, an autologous comparison group provides a controlled environment where complications can be reliably attributed to the technical aspects of the procedure, thus enhancing the validity of the findings.

While the benefits of conducting autologous procedures are manifold, it is also imperative to consider the critical factors involved in developing and employing these control models. This report details the technical aspects and surgical outcomes of two control flaps: an autologous porcine VRAM flap transplant, and the isolation of a neck flap, performed at the same recipient site under conditions of induced ischemia. Autologous VRAM flaps are widely utilized as a versatile reconstructive technique in clinical practice. Therefore, creating a safe and reproducible control model for preclinical VRAM flap allotransplantation studies will not only validate immunological outcomes but also enhance the potential for broader clinical applications of VCA. In contrast, the neck flap with induced ischemia model uses vessel clamping, a standard technique in transplantation surgery, to investigate ischemia-reperfusion injury (IRI) after a specific period of blood flow interruption. This approach facilitates a focused analysis of the physiological responses to IRI while producing minimal to no damage to the flap vessels. A thorough histological evaluation of the flap tissues, complemented by transcutaneous temperature and oxygen measurements, was completed with the purpose of identifying the most effective control procedure for future VRAM flap VCA studies.

## Methods

### Experimental animals

All animals were purchased from Sinclair BioResources. Female 4–5-month-old Yucatan pigs (15.2–17.8 kg) were used for the neck flap surgeries (*n* = 2). Female 3–5-month-old Yucatan pigs (13.2–24.1 kg) were used for the autologous surgeries (*n* = 2). The average overall weight of the experimental pigs was 15.4 ± 2.31 kg (Table 1). All animals were acclimatized for at least five days before surgery. Research was conducted according to the principles outlined in the Guide for Laboratory Animal Facilities and Care prepared by the National Academy of Sciences, National Research Council. The Institutional Animal Care and Use Committee (IACUC) approved the research protocols and living quarters for the animals.

**Table 1:** Characteristics of experimental animals. DOB, date of birth; Neck Flap, neck flap isolation with induced ischemia; Auto, autologous VRAM flap transplantation to the neck.

PIG #	DOB	AGE (mo)	WEIGHT (kg)	SURGERY	COLOR	SEX	BREED
35248	11-2-2022	4.4	15.2	Neck Flap	Pink	F	Yucatan
35247	11-2-2022	5.1	17.8	Neck Flap	Pink	F	Yucatan
36075	9-29-2023	3.6	13.2	Auto	Grey	F	Yucatan
35775	4-30-2023	5.6	24.1	Auto	Pink	F	Yucatan

### Preoperative care

Before surgery, all animals are held nil per os for 12 h. The pigs were anesthetized using ketamine (10 mg/kg) and xylazine (2 mg/kg), given intramuscularly (IM), and maintained under isoflurane following intubation. All procedures were performed under sterile conditions with continuous monitoring of the animal's vitals throughout the surgery. All pigs underwent pain and infection management with 0.12 mg/kg Buprenorphine Extended-Release (Bup ER) given subcutaneously (SQ), 4 mg/kg Carprofen SQ, and 40 mg/kg Cefazolin IV or 5 mg/kg Excede IM. All animals also underwent central line catheter placement as previously described (15).

### Neck flap isolation with induced ischemia

**Figure 1:**
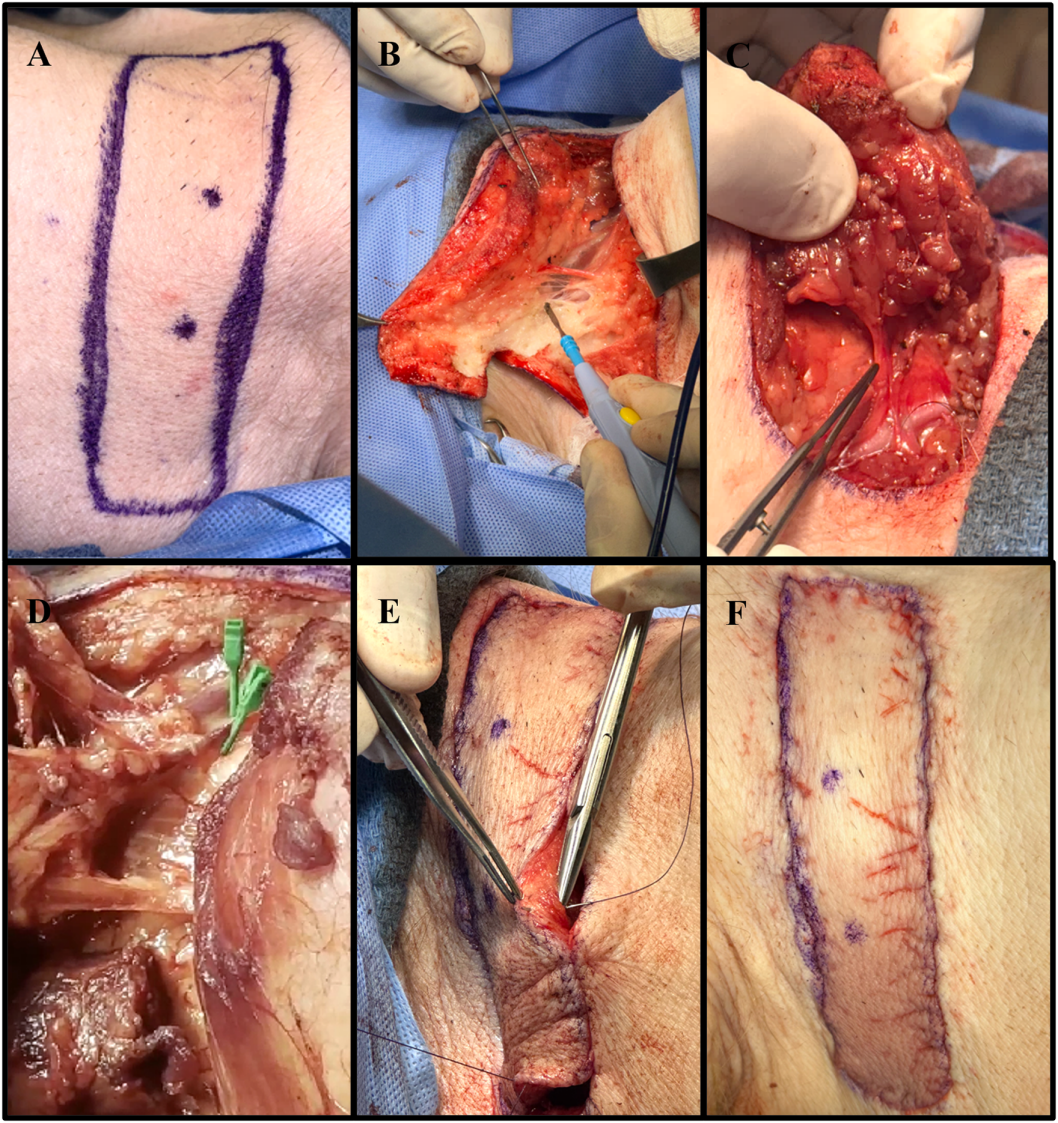
Collective images of the neck flap isolation and induced ischemia procedure. **(A)** Preoperative flap markings on the right anterolateral neck of pig #35247. **(B)** Electrocautery dissection of the subcutaneous tissue portion of the flap. **(C)** Isolation of the main perforator vessels branching from the internal carotid artery. **(D)** Clamping of the two main perforator vessels using Acland clamps. **(E)** Placement of deep dermal sutures to secure the flap into the defect following induced ischemia. **(F)** Fully inset flap at the end of surgery.

Following intubation, the pigs were placed in a lateral decubitus position to access the right anterolateral neck. A horizontal elliptical preoperative marking was made on the side of the neck (Figure 1A). The apex of the flap was placed midline. Procurement of the flap was performed by careful dissection of the skin, subcutaneous tissue, and superficial strap muscles (Figure 1B). The sternocleidomastoid muscle was divided, and dissection continued medially until the main perforating vessels branching from the right internal carotid artery (RICA) and the right external jugular vein (REJV) were identified (Supplementary Material S1). These vessels were carefully isolated (Figure 1C). After the flap was elevated and islanded on its pedicle, Acland clamps were applied to the two main perforating vessels, achieving complete ischemia (Figure 1D). Ischemia was maintained for 20 min for each flap. During this time, the flap was returned to its anatomical position. After the ischemia period, the clamps were released, and the vessels were inspected for any damage that may have occurred secondary to clamping. Once the vessels were deemed stable, with no evidence of injury, the flap was placed back into the lateral neck defect. The deep dermal layer was then closed with an interrupted, inverted 3-0 Vicryl suture, and the skin was reapproximated using a 4-0 Vicryl running subcuticular suture (Figures 1E,F).

### Autologous VRAM flap surgery

**Figure 2:**
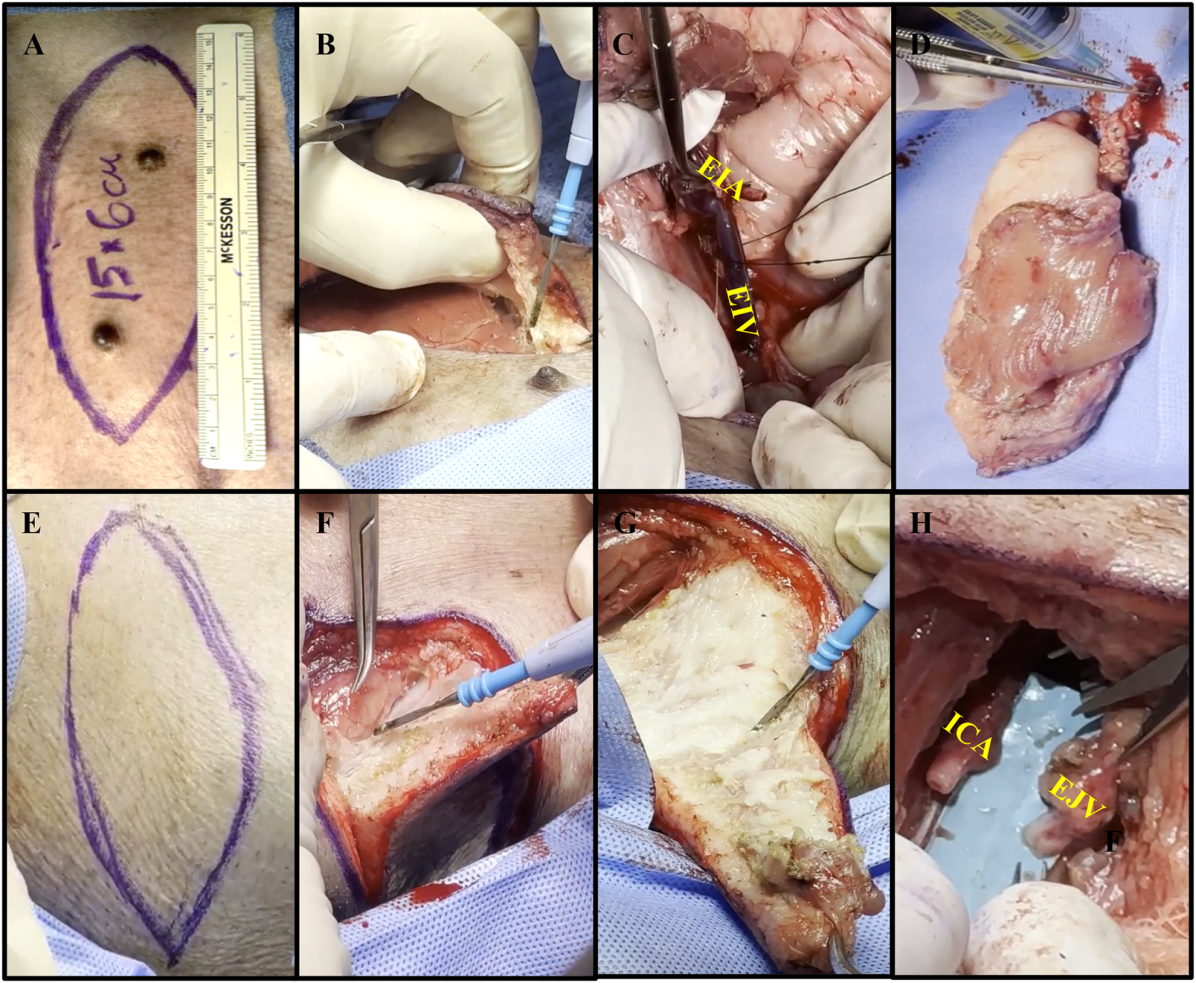
Collective images of the autologous VRAM flap transplant. **(A)** Preoperative markings on pig #35775's left lower abdominal quadrant with a paper ruler indicating flap size. **(B)** Electrocautery dissection through the superficial ventral abdominal muscles, down to the rectus fascia. **(C)** Use of silk ties to divide and harvest the external iliac vein (EIV), to serve as the main vein of the pedicle. External iliac artery (EIA) dissected to the right. **(D)** Procured VRAM flap undergoing ex-vivo heparinized saline flush via the EIA. **(E)** Biconvex surgical marking, slightly smaller than the dimensions of the abdominal flap, on the right anterolateral neck. **(F)** Creation of a soft tissue defect using electrocautery to carefully dissect the subcutaneous tissue. **(G)** Further dissection of the neck flap using electrocautery. **(H)** Isolation of the internal carotid artery (ICA) and external jugular vein (EJV) over 5 cm, with proximal clamping.

The method of procuring each autologous flap was performed as previously described for an allogenic VRAM flap based on the external iliac vessels (15). In brief, the VRAM flap is designed in a lenticular shape measuring roughly 15 × 6 cm and is procured from the left lower abdominal quadrant, capturing the nipples (Figure 2A). The skin is incised, and electrocautery dissection is performed through the superficial ventral abdominal muscles down to the rectus fascia (Figure 2B) (Supplementary Material S2). Dissection continues down into the pelvis, and the rectus muscle is separated from the peritoneum and elevated with the flap. The external iliac vessels are identified and dissected distal to the bifurcation of the internal iliac vessels, where the left external iliac artery (LEIA) and vein (LEIV) are divided with surgical clips or silk ties (Figure 2C). The flap, containing the pedicle, is then removed from the abdominal cavity and intravascularly flushed with 50–100 ml of normal saline containing heparin sulfate (1,000 IU/L) (Figure 2D). The warm ischemia time was between 50 and 90 min for each flap.

**Figure 3:**
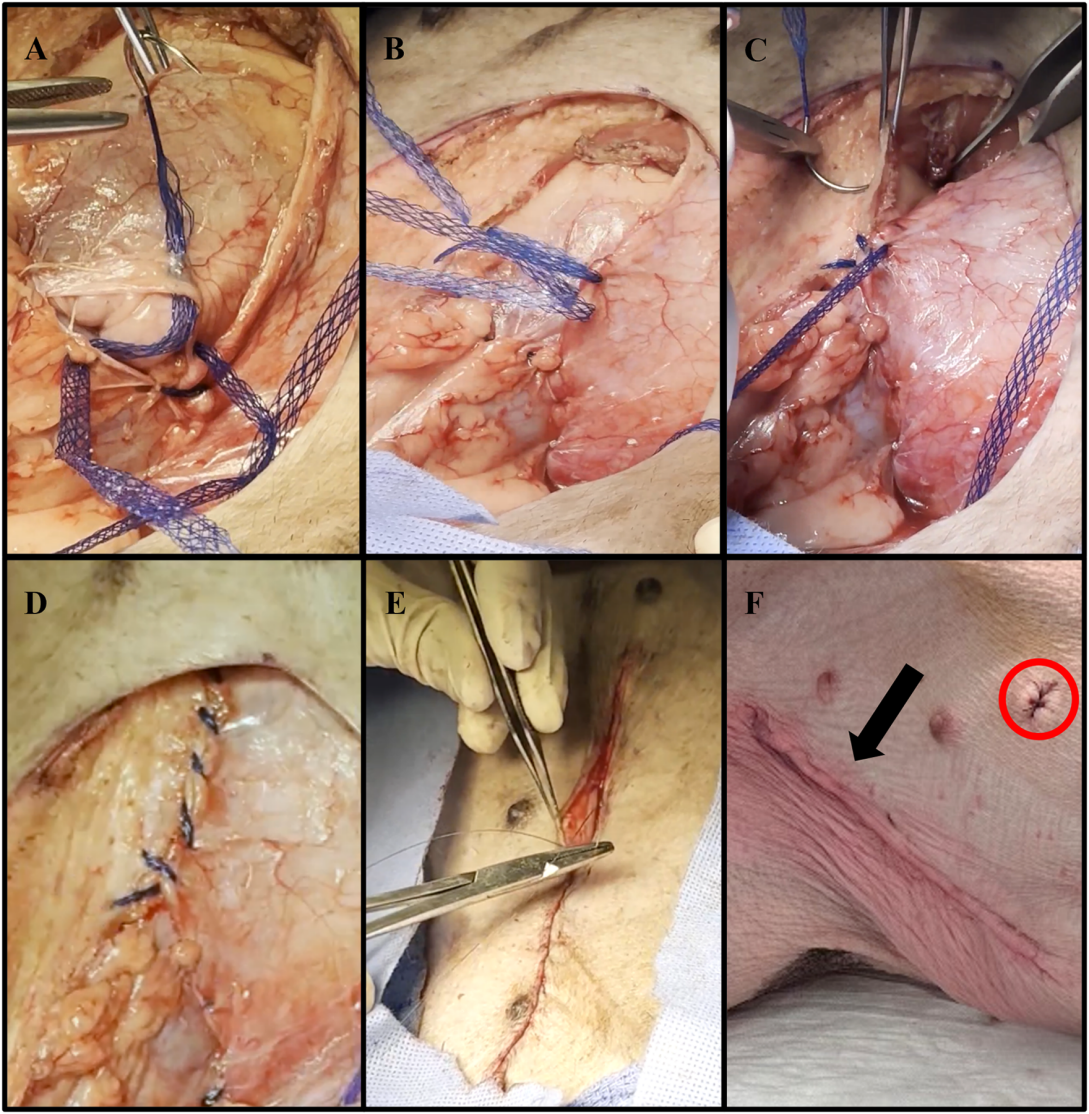
Representative images of abdominal wall defect closure using DURAMESH™ Mesh Suture (Chicago, IL) following autologous VRAM flap procurement. **(A)** Lateral to medial closure of the parietal peritoneum defect using DURAMESH™. **(B)** Placement of continuous over and over sutures within the Scarpa fascia in a caudal to cranial fashion. **(C)** Successful re-approximation of the Scarpa fascia. **(D)** Complete low-tension closure of the Scarpa fascia. **(E)** Closure of the abdominal skin using a continuous nonabsorbable 4-0 Vicryl running subcuticular suture. **(F)** Postoperative image of closed left lower abdominal wound (black arrow). The sutured area (red circle) represents where biopsies were taken for histological analysis.

The abdominal defect created from the VRAM flap procurement was closed using size 1 (4 metric) DURAMESH™ Mesh Suture (Chicago, IL). The parietal peritoneum opening was closed in a lateral to medial fashion using the suturable mesh (Figure 3A). Continuous over and over sutures were placed within the Scarpa fascia in a caudal to cranial fashion (Figure 3B) to successfully re-approximate the tissue (Figures 3C,D). Complete closure of the abdominal skin was achieved using a continuous nonabsorbable 4-0 Vicryl running subcuticular suture (Figures 3E,F). The abdominal wound was then covered with Tegaderm and left open to air starting on postoperative day (POD) 1.

Following abdominal flap procurement and wound closure, the animals were placed in a lateral decubitus position to access the right anterolateral neck. After placement of a contraleral (left) central line catheter, a horizontal lenticular shape was outlined on the right neck (Figure 2E). A composite soft tissue defect was created by excising skin, subcutaneous tissue, and superficial strap muscles, just smaller than the size of the flap (Figures 2F,G). Dissection was continued through the sternocleidomastoid muscle, brachiocephalicus, and sternocephalicus muscle until the RICA and REJV were identified. After the removal of the neck tissue and identification of the RICA and REJV, the vessels were carefully isolated and dissected for 5 cm, then proximally clamped in preparation for microvascular anastomosis (Figure 2H).

### Microsurgical anastomosis

**Figure 4:**
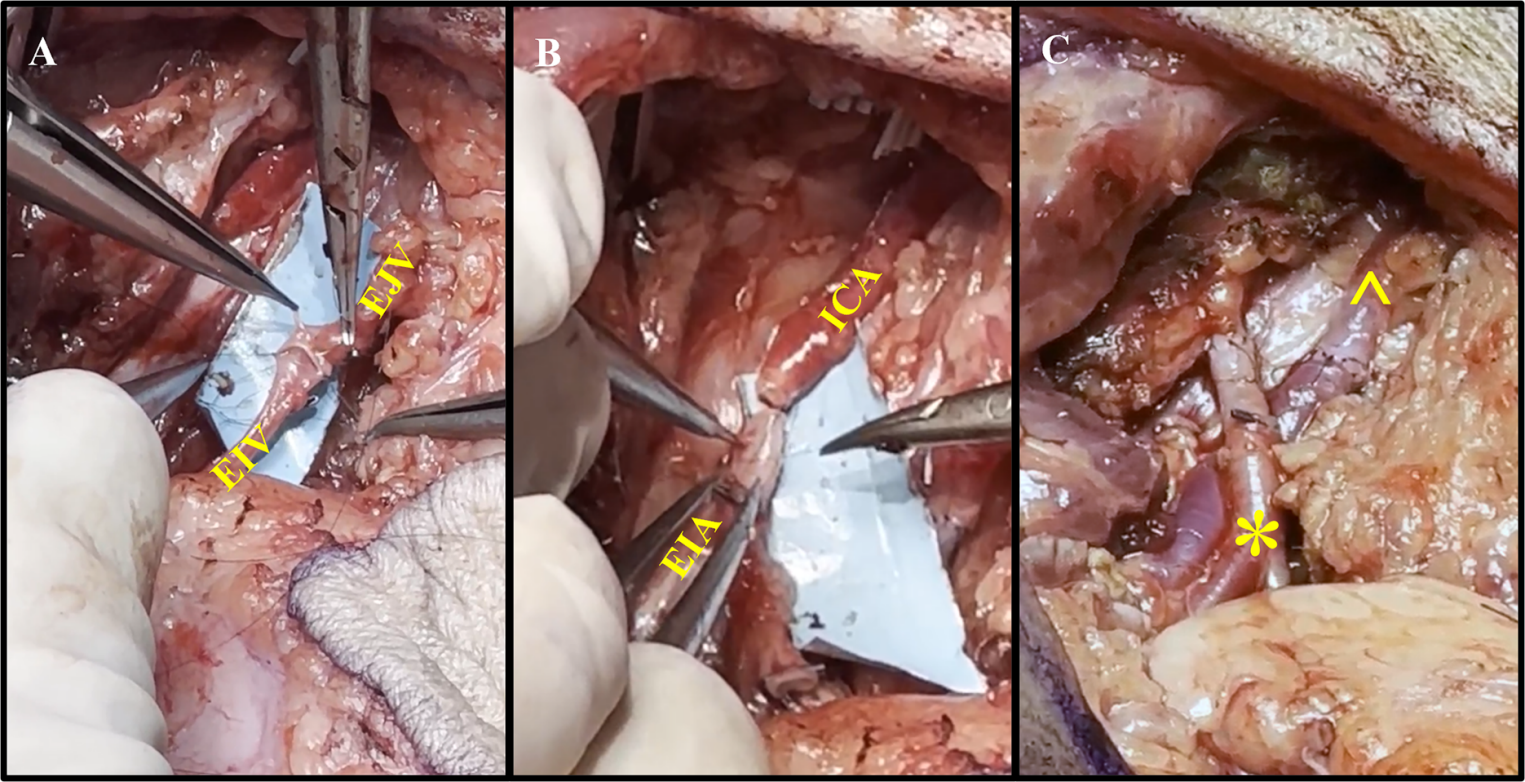
Representative process of vessel anastomoses. **(A)** Anastomosis of the external jugular vein (EJV) to the external iliac vein (EIV) using end-to-end suturing. **(B)** Anastomosis of the internal carotid artery (ICA) and external iliac artery (EIA) using end-to-end suturing. **(C)** Completion of the vascular (*) and venous (^) anastomoses without signs of anastomotic leakage.

**Figure 5:**
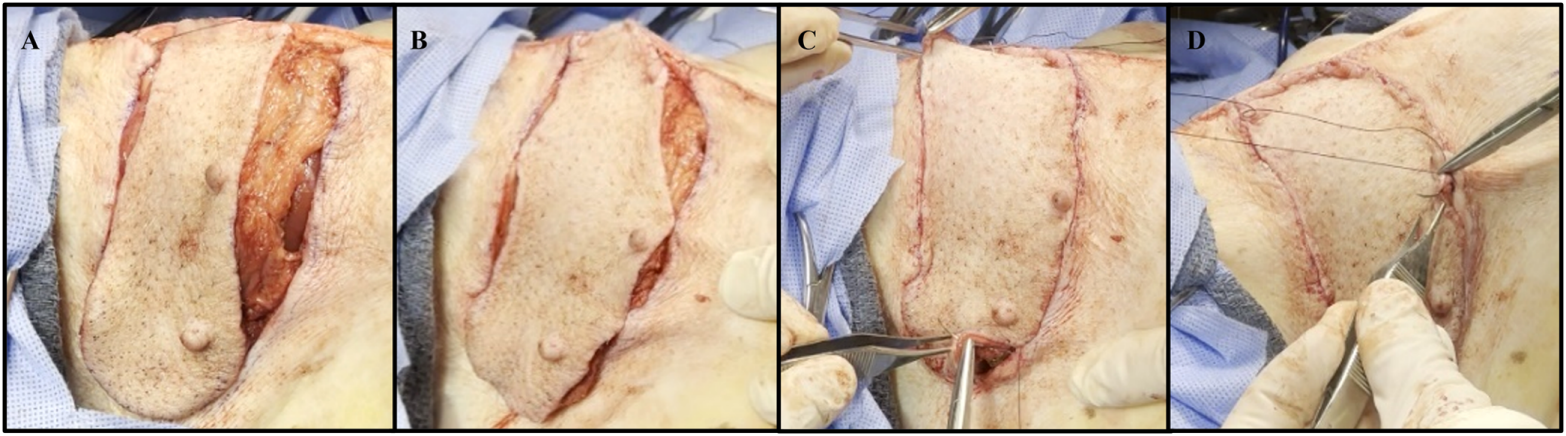
Representative images of the autologous VRAM flap transplantation to the anterolateral neck defect. **(A)** Autologous flap inset on the right neck of pig #35775. **(B)** Approximation of the autograft skin to the neck skin by placing 3-0 interrupted simple sutures at multiple tension points. **(C)** Placement of circumferential 3-0 interrupted simple sutures. **(D)** Complete closure using a continuous nonabsorbable 4-0 Vicryl running subcuticular suture.

The method of microsurgical anastomosis of the VRAM flap pedicle vessels to the neck vessels was performed as previously described^16^. In brief, the VRAM flap pedicle was placed into the neck tissue defect and all vessels were prepared for anastomosis by visual inspection, excision of adventitia, and irrigation with heparinized saline. Using at least 3.5X magnifying surgical loupes, the LEIA and LEIV are hand-sewn, end-to-end, to the RICA and REJV, respectively, using 9-0 nylon interrupted sutures (Figures 4A–C) (Supplementary Material S3). Proximal clamps are then removed to analyze the quality of the anastomoses and once it is deemed satisfactory, the flap is inset, and a fenestrated Penrose drain is placed. The deep dermal layer is then closed with an interrupted, inverted 3-0 Vicryl suture, and the skin is reapproximated using a 4-0 Vicryl running subcuticular suture (Figures 5A–D).

### Postoperative care

The neck of each pig was wrapped with two layers of McKesson tubular elastic retainer net (size 7), to protect the wound. Food and water were reinstituted immediately after each animal recovered from surgery. All flaps were monitored daily via physical examination (palpation, tactile warmth assessment, and visual inspection). The integrity of the anastomosis of each flap was assessed by visual inspection of flap color as well as the presence of bleeding following daily punch biopsies. The animals were also followed for any fluid collections (seromas/hematomas) around the flap, which were drained percutaneously if clinically significant.

### Histology

Tissue punch biopsies (6 mm) were collected postoperatively at predetermined sequential time points. Biopsies were geographically spaced to prevent non-specific inflammation due to biopsy site reactions. Biopsies of normal abdominal tissue, neck flap tissue, and autologous flap tissue were collected and fixed immediately in 10% formalin. Samples were then embedded in paraffin and sectioned (5 um) for hematoxylin and eosin (H&E) staining. Evaluation of inflammation and edema was performed by a transplant pathologist without knowledge of the tissue source.

### The oxygen and temperature sensor

The oxygen and temperature sensor used in this study was closely based on a previously published design (16). It includes an electronic reader and oxygen-responsive material. The sensor operates through the principle of phosphorescent quenching, where the phosphorescence lifetime of a sensor molecule is inversely proportional to the partial pressure of oxygen (pO2) via collisional interactions. For example, at high oxygen concentrations, the lifetime of the phosphorescence is short, meanwhile the phosphorescence lifetime increases in the setting of decreasing pO2. Phosphorescence lifetime can be calibrated and related to pO2 via the Stern-Volmer equation (17).

The oxygen sensing material uses a highly sensitive, ultrabright metalloporphyrin molecule (18) embedded within a polypropylmetharcylate (PPMA) polymer material. This porphyrin-PPMA material is packaged within a film composed of an atmosphere-facing layer comprised of Bioclusive polyurethane dressing material and a skin-face layer comprised of breathable silicone impregnated with titanium dioxide. The material was developed to be moisture and humidity-insensitive and has been previously tested in both animal and human studies (16, 18–20). The atmosphere-facing layer serves to insulate the porphyrin-PPMA material from atmospheric oxygen, while the skin-facing layer isolates the sensor from the skin and improves the amount of collected light via backscattering. Prior testing of this material has found it does not leach porphyrin into the skin at levels detectable via inductive plasma coupled mass spectroscopy analysis (21).

**Figure 6:**
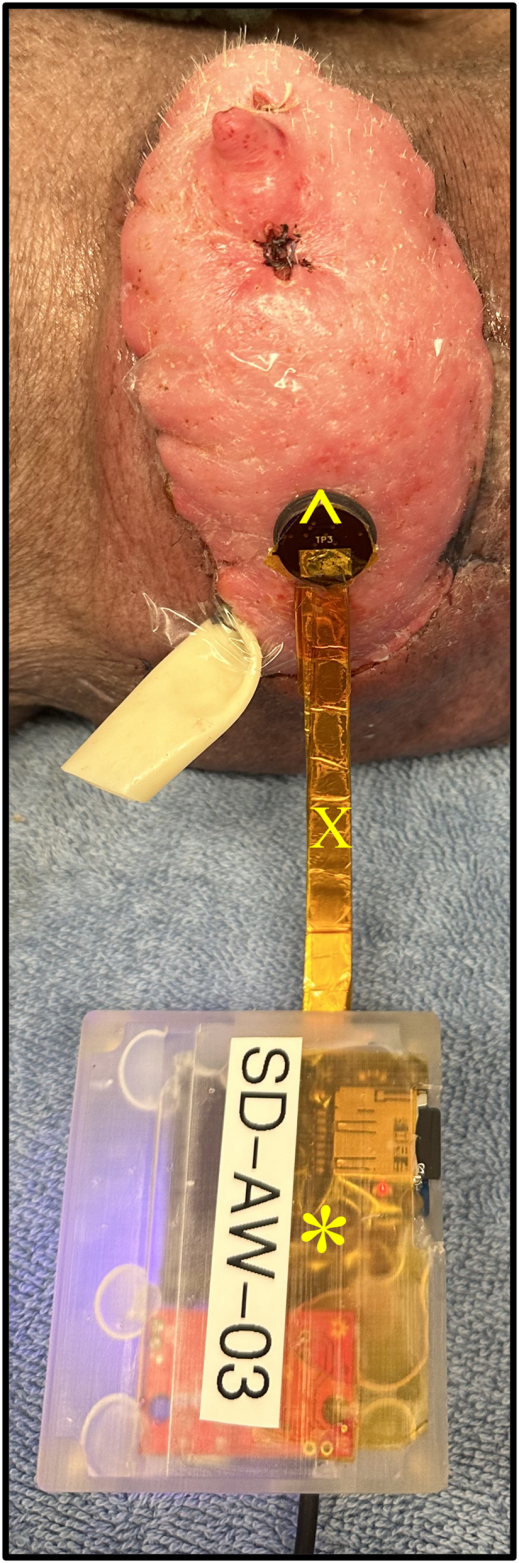
Transcutaneous oxygen and temperature sensor placed flush to a representative graft skin. The sensor is comprised of both a reader head (^) and body (*). The head is connected to the body by a ribbon cable (X).

The electronic phosphorescence lifetime reader was designed to be stand-alone, battery-powered, and self-contained for mobile detection and recording of transcutaneous partial oxygen pressure. The reader is comprised of both a reader head and body, where the head is made to adhere to the sensor material and is connected to the body by a ribbon cable (Figure 6). The reader's head excites the sensor material via two 385 nm LEDs and the subsequent phosphorescence emission is collected with a photodiode. A thermistor is additionally positioned on the sensor head to measure skin temperature. The body of the sensor is based on a Particle Photon Arduino microcontroller mounted on a custom circuit board. To record oxygen and temperature data, an Adafruit secure digital (SD) card reader was added to the circuit board. The sensor runs custom firmware written in C that orchestrates phosphorescence lifetime measurements through a sinusoidal modulation/modulo operator method (16) and records the oxygen partial pressure and temperature data on the SD card.

Sensor materials and readers were calibrated using a custom-developed calibration device and protocol. A programmable gas mixer was used to precisely deliver known air/nitrogen gas ratios over time, and a heating element was used to automatically change the temperature experienced by the sensor and material. This allowed the sensors to be calibrated over a large range of oxygen partial pressures and temperatures, which was found to be critical for the successful operation of the device. Analysis and calibration of the sensor were accomplished using a custom Python (22) program that calculated the phosphorescence lifetime, translated the values to pO2, and exported the data into CSV files. In this study, the sensors were placed either on the graft site or on an abdominal (inguinal) control site at predetermined sequential time points following surgery. The abdominal control location was selected as the skin is relatively thin, allowing for high oxygen transport for routine oxygen detection and measurement.

### Oxygen and temperature data analysis

Analysis and plotting of the oxygen partial pressure and temperature data were carried out using R and RStudio (23). A custom R notebook was written to explore, statistically analyze, and plot the data. The difference between temperature and pO2 was calculated between the graft and respective control for each day and each pig. While linear mixed effects analysis was initially attempted via the LME4 package (v1. 1-26; Bates et al., 2015), it was observed that the random effect variable (pig number) had essentially zero variance, leading to a singular fit. To avoid this, a partial Bayesian method was instead employed via the BLME package (v1. 1.0-5; Chung, et al., 2013) that made use of regularizing priors to avoid singularity (24). The replant vs. transplant status of the animal was included as a nested parameter. Both oxygen tension and temperature were tested using this function call in R: blme[value∼fixed_effects + (1|replant:pig_num)].

Fixed effects can include a day numeric value following surgery, a binary level representing if the surgical procedure was a neck flap (1) or autologous transplant (0), and an interaction term between day and procedure type. Calculation of *p* values was accomplished using the parameters: *p*_value() function and values ≤0.05 were considered statistically significant. R interaction plots were used to determine if an interaction term was included in the analysis. For example, if the plotted values of the neck flap and transplant were observed to either not be parallel or cross, it was determined that an interaction term was included.

## Results

### Surgical time

The average flap procurement time for the neck flap isolation with induced ischemia procedure was 50.5 ± 14.9 min and the average abdominal flap procurement time for the autologous transplantation was 120 ± 56.6 min (Table 2). The average flap transplant time was 115 ± 21.2 min. The total surgery time for both the neck flap and autologous transplant groups was 135 ± 49.5 and 278.5 ± 47.4 min, respectively. Total surgical time includes the sum of flap procurement time, transplant/ischemia time, and additional procedures such as central line placements, and closure of incisions (Table 2). The warm ischemia time was between 50 and 90 min for each autologous flap.

**Table 2:** Total surgical times for each procedure including flap procurement time and transplantation/ischemia time.

Surgery	Pig Number	Flap Procurement Time (min)	Average Flap Procurement Time (min)	Transplant Time/Ischemia Time (min)	Average Flap Transplant Time/Ischemia Time (min)	Total Surgical Time^a^ (min)	Average Total Surgical Time^a^ (min)
Neck Flap	35248	61	50.5 ± 14.9	20	20	170	135 ± 49.5
Neck Flap	35247	40		20		100	
Auto	36075	80	120 ± 56.6	100	115 ± 21.2	245	278.5 ± 47.4
Auto	35775	160		130		312	

^a^ Total surgical time includes additional procedures such as central line placements and closure of incisions. Neck Flap, neck flap isolation with induced ischemia; Auto, autologous VRAM flap transplantation to the neck.

### Autologous VRAM flap transplant

**Figure 7:**
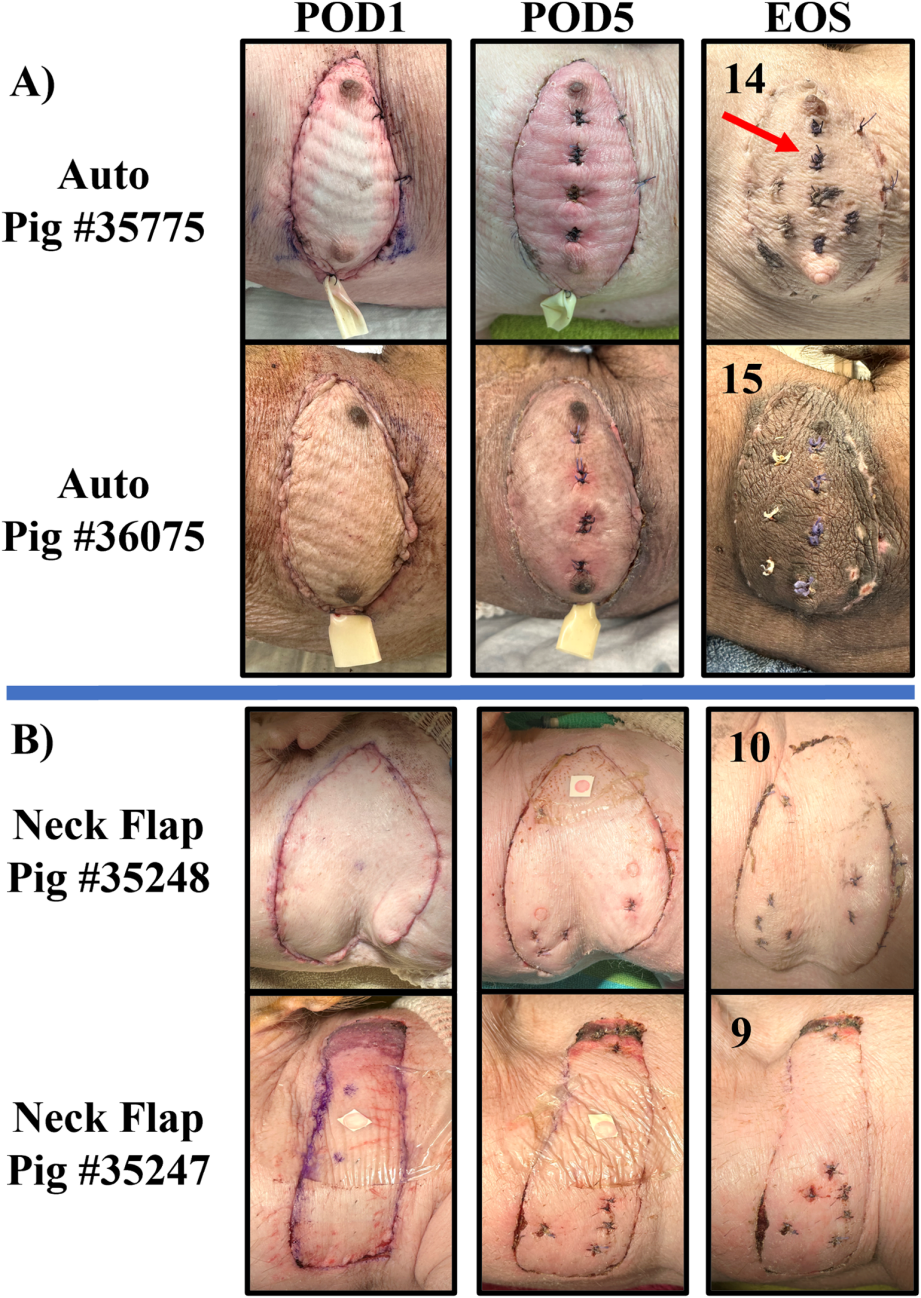
Representative collection of graft images taken on POD 1, 5, and at the end of the study (EOS) for the autologous VRAM flap transplant (Auto) group **(A)** and the neck flap isolation and induced ischemia (Neck Flap) group **(B)**. The EOS POD is indicated in the top left corner of each image. The neck flap group did not require the placement of Penrose drains due to a lack of postoperative edema. The sutured areas (red arrow) represent places where prior biopsies were taken for histological analysis.

Two autologous flaps were successfully transferred from the left abdomen to the right anterolateral neck of two separate pigs, with effective closure of abdominal defects using suturable mesh. Both pigs experienced a return to normal gait immediately following surgery. Each flap demonstrated good perfusion of both the skin and the muscle following vessel anastomosis. Consistent perfusion of each autograft was observed throughout the entire postoperative period. This was demonstrated by healthy flap color, warm temperature via palpation, progressive healing, and bleeding following punch biopsies. On POD1, on average, there was minimal erythema, and the flaps continued to show good color and signs of adequate blood flow to the skin (Figure 7A). On POD5, mild erythema was observed in both animals, involving the entire flap surface (Figure 7A). Both flaps were fully healed by POD7 (data not shown). We followed the healing process of the flaps for up to 15 days before the animals were euthanized. At the end of the study, there was evident incorporation of the flaps into the lateral neck tissue with no signs of erythema, edema, or necrosis (Figure 7A).

### Neck flap isolation with induced ischemia

Two right anterolateral neck flaps were successfully elevated on two separate pigs and survived 20 min of temporary ischemia. No damage to the pedicle vessels was observed following clamping. Consistent perfusion of each flap was observed throughout the entire postoperative period, evidenced by a healthy flap color, warm palpable temperature, progressive healing, and bleeding after punch biopsies. Gross inflammation and edema were minimal to absent in both flaps throughout the postoperative period (Figure 7B). Both neck flaps were fully healed by POD7 (data not shown) (Figure 7B). The healing process of both flaps was monitored for up to 10 days before the animals were euthanized. At the end of the study, there was evident reincorporation of the flaps into the lateral neck tissue.

### Complications

**Figure 8:**
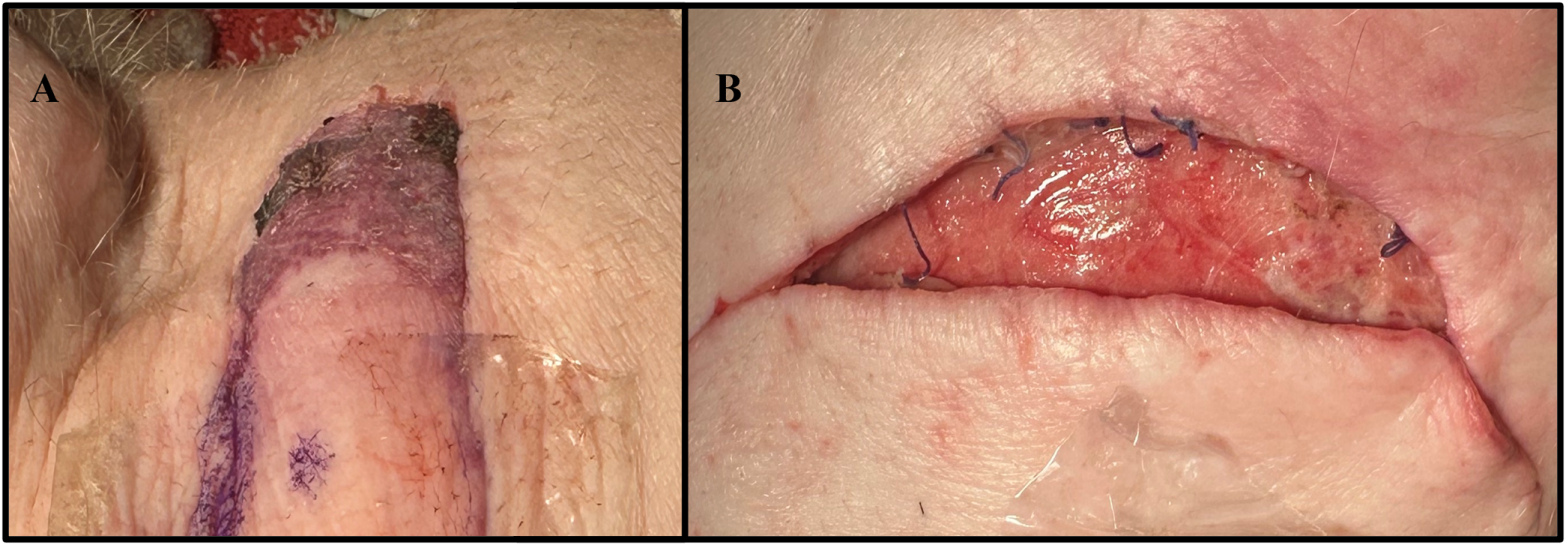
Complications experienced by the neck flap isolation with induced ischemia cohort. **(A)** Dorsal flap necrosis in Pig #35247 on POD2. **(B)** Caudal wound dehiscence in Pig #35248 on POD2.

All animals recovered from surgery with demonstrated ambulation and appetite immediately following each procedure. No animals died during surgery or from surgery-related complications. There were also no occurrences of anesthesia-related events, flap loss, hematomas, seromas, or infections in either group. No postoperative complications including hernias, bulges, or functional deficits, were experienced in the autologous group. However, in the neck flap group, one flap presented with signs of necrosis in the dorsal area on POD1, and the other experienced wound dehiscence on POD2, requiring reoperation (Figure 8).

### Histological evaluation

**Figure 9:**
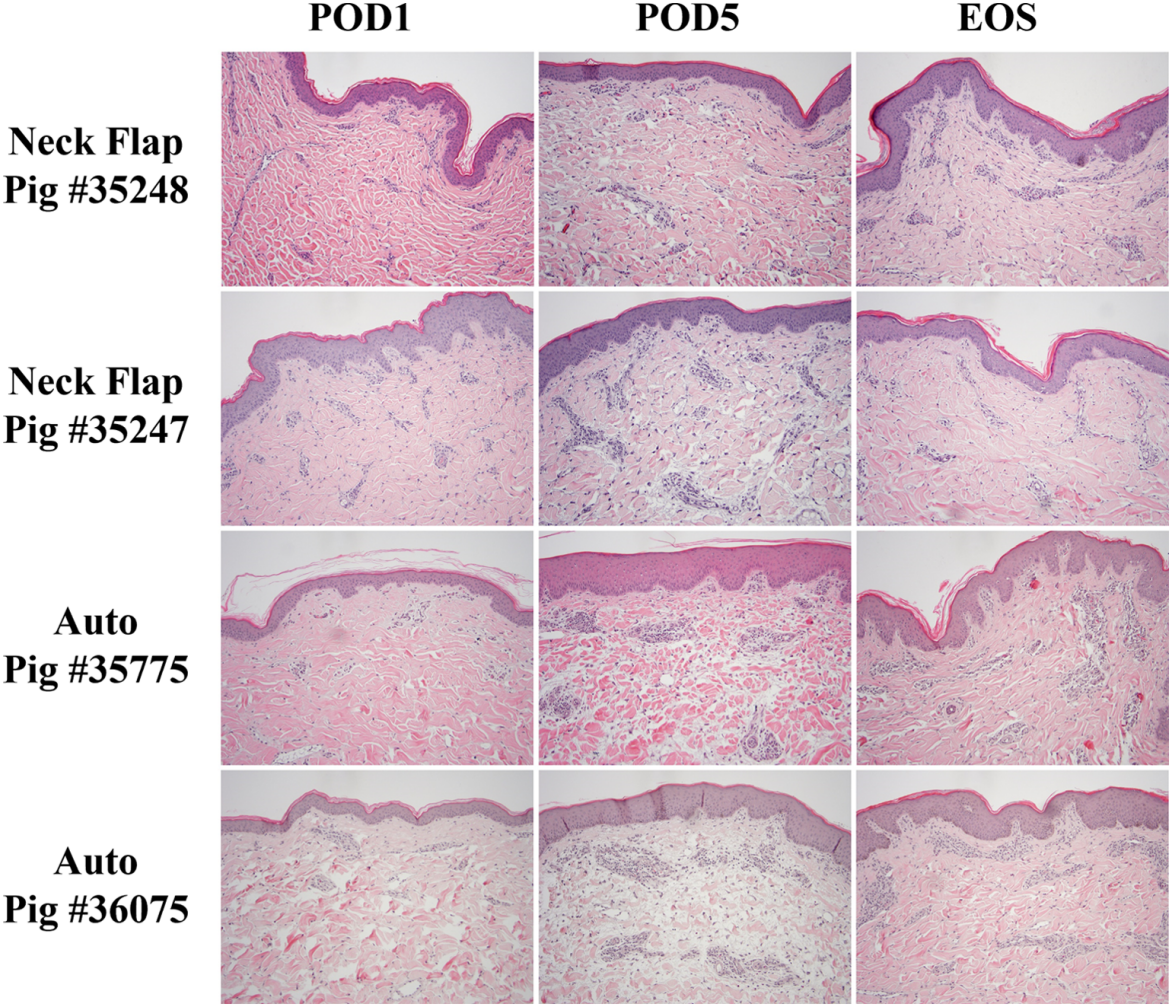
Representative histology comparing the neck flap isolation and induced ischemia (Neck Flap) group to the autologous transplants (Auto) on POD1 and 5 and at the end of the study (EOS). Both neck flaps showed only mild dermal edema on POD5, resolving by the EOS. Both autologous transplants showed mild to moderate dermal edema and mild dermal perivascular inflammation on POD5, with resolution of the edema and partial resolution of the inflammation by the EOS. The EOS POD is indicated in the top left corner of each image. All Hematoxylin & Eosin images are at 100x and equally white-balanced.

The neck flap group showed, on average, only mild dermal edema on POD5, which resolved by the end of the study (Figure 9). In contrast, the autologous transplants demonstrated, on average, slightly greater dermal edema and dermal perivascular inflammation on POD5, with resolution of the edema and partial resolution of the inflammation by the end of the study. In this group, there was mild eosinophil-rich perivascular inflammation that peaked on day 5 (Figure 9), suspicious of a drug reaction or contact dermatitis. However, there was no evidence of arteritis or epidermal inflammation in the autologous grafts at any time point (Figure 9).

### Transcutaneous oxygen and temperature

**Figure 10:**
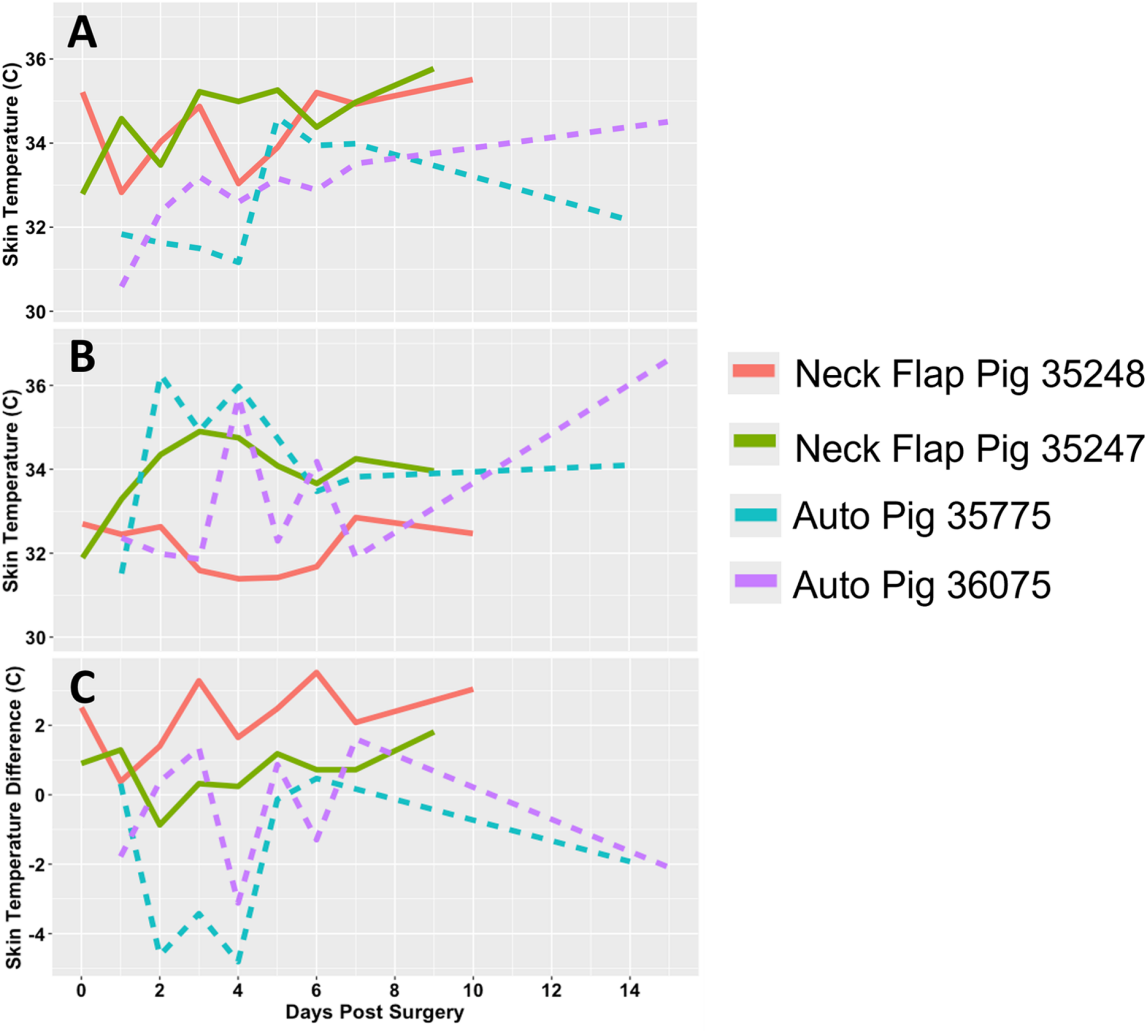
Transcutaneous temperature readings. **(A)** Temperature (°C) measured from the flap of each experimental pig. **(B)** Temperature (°C) measured from the abdominal control skin of each experimental pig. **(C)** Difference in temperature (°C) between each graft and their respective control site. Each animal is represented by a different line color with the autologous grafts illustrated by dotted lines and the neck flaps with induced ischemia represented by solid lines. Neck Flap; neck flap isolation with induced ischemia, Auto; autologous VRAM flap transplantation to the neck.

The oxygen and temperature sensor was used at daily intervals following the neck flap and autologous transplant procedures. The temperature measurements of the abdominal skin control sites were observed to be relatively consistent across all animals with a mean surface temperature of 33.4°C (Figure 10B). In contrast, the temperatures of both the autologous and the neck flaps were observed to rise in the postoperative period (Figure 10A); this increase was found to be statistically significant across all animals using a partial Bayesian linear mixed effects analysis comparing graft temperature and day post-surgery (*p* = 0.015). Temperature was also found to be predicted by the type of surgical procedure (neck flap vs. autologous transplant, *p* = 0.037). The difference in temperature between grafts and their respective controls (Figure 10C) was not observed to be statistically significant.

**Figure 11:**
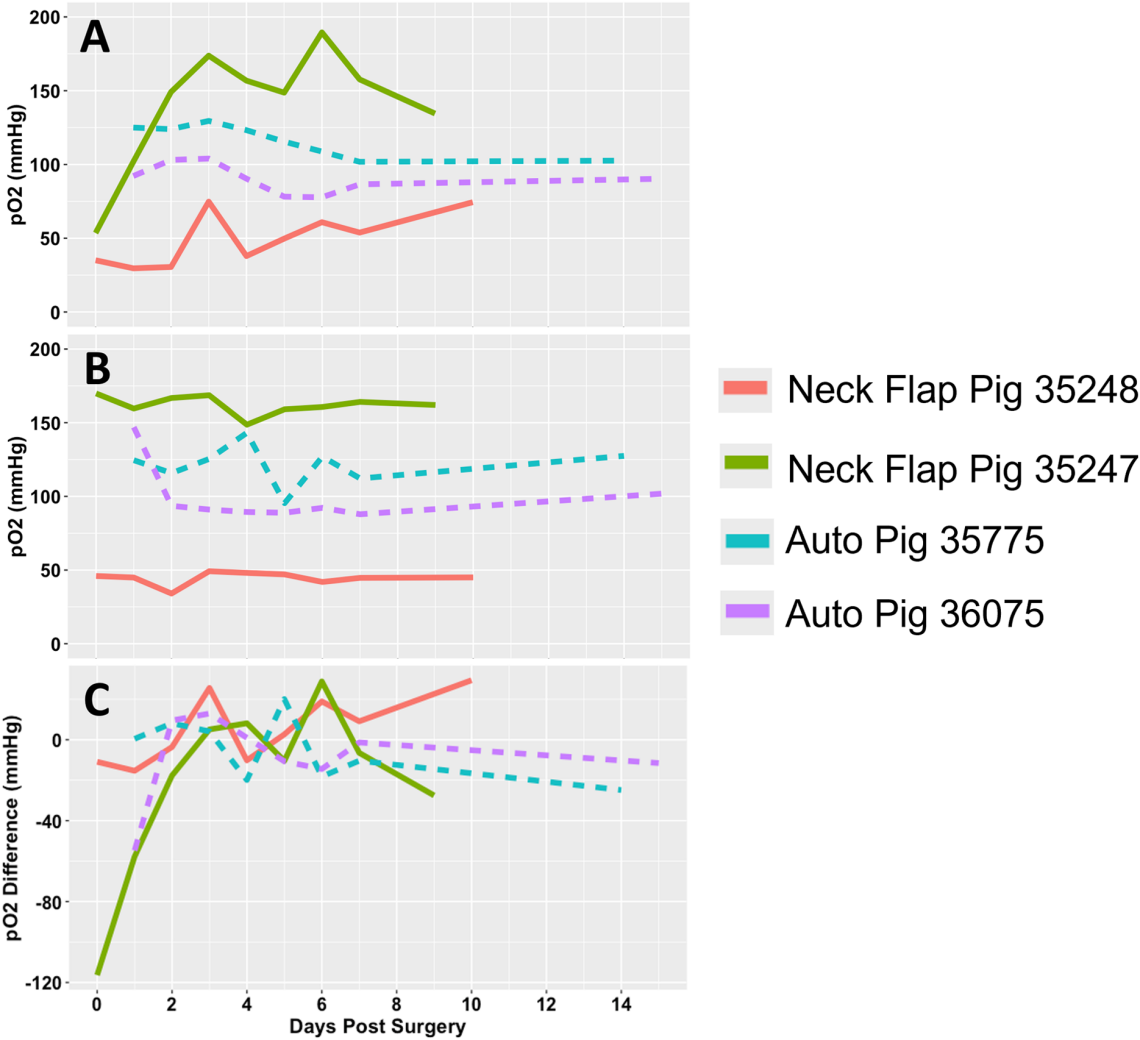
Transcutaneous oxygen tension over time. **(A)** Partial pressure of oxygen (pO2) readings measured from the flap of each pig. **(B)** pO2 readings measured from the abdominal control skin of each pig. **(C)** pO2 difference between each graft and their respective control site. Each animal is represented by a different line color with the autologous grafts illustrated by dotted lines and the neck flaps with induced ischemia represented by solid lines. Neck Flap; neck flap isolation with induced ischemia, Auto; autologous VRAM flap transplantation to the neck.

There was a relatively wide range of transcutaneous pO2 measured from both the graft and control sites in the days following both procedures (Figures 11A,B). The pO2 measured in the autologous transplants flaps was relatively stable beyond POD1, meanwhile, the neck flaps demonstrated an increase in pO2 over time (Figure 11A). The difference in pO2 between the graft and control sites demonstrated time-dependent differences in both the initial rise in pO2 following surgery as well as their trends over time (Figure 11C). A partial Bayesian linear mixed effects analysis found the procedure-dependent change in pO2 over time to be statistically significant for both the graft pO2 values (*p* = 0.002) as well as the graft-control pO2 differences (*p* = 0.011).

## Discussion

In the present study, we utilized a recently established porcine VRAM flap model (15) to compare the surgical outcomes of both autologous VRAM flap transplantation and lateral neck flap isolation with induced ischemia. Swine provide an ideal animal model for VCA research due to their anatomical and physiological similarities to humans (25–27). In particular, miniature pigs are more manageable for extended flap observation periods and exhibit anatomical structures and immunological responses that closely resemble those of humans (28, 29). For example, porcine skin is structurally, cellularly, and antigenically similar to human skin (30). As a result, transcutaneous oxygen and temperature data obtained from these animals are more reliable and translatable to humans. The VRAM flap is composed of skin, subcutaneous fat, muscle, and vasculature making it a valuable model for the evaluation of immunologic outcomes. The advantages of this flap include a substantial amount of tissue to fill large tissue defects and a wide degree of rotation for flap positioning. Furthermore, this myocutaneous flap contains a large adipose compartment which provides ample adipocutaneous perforators for the transcutaneous investigation of oxygen and temperature to monitor flap status (31).

Autologous graft transplants, where tissue is taken from an animal and transplanted back to the same animal, can serve as a valuable control group in VCA studies to define the postoperative inflammation course relative to the onset of rejection, helping to differentiate between complications caused by immune rejection, the surgery itself, or inherent properties of the tissue. However, studies involving porcine VRAM flap models often neglect to include control groups (31–34). Complications associated with donor sites and concerns over the possibility of living donors have impeded the broader use of autologous VRAM flap procedures. For instance, procuring this flap can lead to ipsilateral abdominal wall weakness, which increases the risk of abdominal wall dehiscence and incisional hernias (35). These complications can complicate recovery, affect study outcomes, and impact animal welfare. Given these considerations, it is notable that we successfully completed two autologous VRAM transplants with low-tension abdominal wound closures using suturable mesh. Interestingly, it has been found that wound dehiscence is more likely when there is increased wound tension and incisional hernias can be mitigated by using mesh to reinforce the abdominal wall (35). Additionally, the tension required to close a standard wound is greater on the back of the pig than on its abdomen (36), and vertical flaps, such as the VRAM flap, retract mostly across their width rather than their length (37). In this study, repair of the defect at the flap procurement site with suturable mesh minimized donor site morbidity, as evidenced by a return to normal gait immediately following surgery and a lack of complications throughout the study period. Furthermore, the right anterolateral neck was selected as the recipient site for both procedures because the anatomy of the RICA and REJV is highly preserved, easily accessible, and similar in caliber to the external iliac vessels (15). This location also minimized the ability of the pig to contact the flap, thereby reducing the risk of infection and self-induced graft injuries (15), allowing for convenient daily monitoring through gross examination, tissue biopsies, and transcutaneous measurements. Overall, the success observed in our autologous cohort can be attributed to several critical factors: surgical technique, the choice of flap type, the donor site location, and the application of suturable mesh for abdominal wall support. Consideration of these variables can enhance the efficacy and reliability of future autologous procedures, allowing autologous control groups to become more common in the field of VCA.

To mimic ischemia-reperfusion injury and the inflammation experienced by transplanted grafts, we performed neck flap isolations with induced ischemia. Our observations revealed that after 20 min of ischemia, there was minimal to no gross inflammation or edema in the postoperative period. Histologically, only mild dermal edema was present on POD5. In contrast, the autologous transplant group exhibited noticeable erythema, which began mildly on POD1 and intensified through POD5. Histological examination of this group showed slightly greater dermal edema and mild dermal perivascular inflammation by POD5. Given that VCA grafts have been observed to experience an inflammatory response (damage response) and an acute rejection phase on POD2 and POD5, respectively (38), the autologous transplant more closely mimics the postoperative inflammatory course of these grafts. Additionally, acute rejection in VCA grafts often begins between POD5 and POD6 (38). This corresponds with the period during which we observed the resolution of edema and inflammation in the autologous transplants, both histologically and grossly. This observation is particularly significant as it suggests that the postoperative inflammatory response in the autologous control group does not overlap with the typical onset of rejection. Consequently, this allows researchers studying VCA grafts to more effectively differentiate between inflammation and edema induced by surgical injury vs. that caused by immune rejection. Furthermore, a notable advantage of VRAM flaps is the amount of surface area available for substantial biopsies with a broader biopsy core. This flap model allowed for a more complete histological sampling and analysis of larger arteries in the subcutaneous and deep dermis layers, without compromising the integrity of the flap.

All of the neck flaps with induced ischemia experienced postoperative complications, which included dorsal flap necrosis and wound dehiscence. These issues may have resulted from technical errors, such as damage to the blood supply during flap dissection or excessive tension during flap closure (39). Alternatively, the anterolateral neck region lacks well-defined perforating vessels. Therefore, with only a limited presence of small perforators, the procurement process may lead to a degree of flap loss, even when the technical aspects of the harvest are executed successfully. In contrast, the autologous cohort did not experience any postoperative complications, despite arguably undergoing a more complex procedure. The absence of complications in this group could be attributed to a more robust blood supply, owing to the vertical orientation of the VRAM flap, which optimizes the number of perforating vessels supplying the skin paddle (40). Additionally, the flap was designed to exceed the defect size in length and width, allowing it to cover the defect with minimal tension. This flap-to-defect size mismatch may have provided protection against wound dehiscence and overstretching. In comparison, the neck flap procedures involved flaps that were matched precisely to the defect size, thereby increasing the risk of tension during closure. The neck flap group also exhibited a shorter average total surgical time compared to the autologous transplants (135 ± 49.5 min vs. 278.5 ± 47.4 min, respectively). Previous research indicates that the average surgical time for an allogeneic VRAM flap, based on the external iliac vessels, is approximately 291.5 ± 9.19 min (15). This suggests that the autologous transplant model more closely aligns with the duration of allogenic procedures, thereby reducing variability in critical factors such as surgical invasiveness (41), tissue exposure to contamination (42), and the duration of anesthetic administration (41). Matching surgical durations ensures that all interventions are exposed to comparable physiological conditions, thereby minimizing potential confounding variables and enhancing the reliability of study outcomes.

Interestingly, we observed that transcutaneous temperature readings increased in the days following surgery for both groups. Further analysis revealed that this temperature rise was influenced by the type of surgical procedure. Specifically, the neck flap group exhibited both an initially higher temperature and a statistically significant rate of temperature increase compared to the autologous transplants (*p* = 0.015). This discrepancy may be attributable to the nature of the skin used in each procedure. The neck flap procedure involved mobilizing and repositioning native neck tissue, while the autologous procedure involved transferring abdominal tissue to the lateral neck. Differences in the volume of tissue and skin thickness between the grafts could also account for the observed variations in temperature and its changes postoperatively. Moreover, a decrease in skin surface temperature may follow free flap transfer due to the significant alterations in flap physiology. Unlike normal skin, free flaps are stripped of afferent and efferent nerve connections, leading to a hemodynamically significant sympathectomy (43). Consequently, flaps like the VRAM flap are disconnected from both their native neural network and central thermoregulation mechanisms, which may cause abnormal transcutaneous temperatures, particularly during the healing process.

The pO2 values recorded from the abdominal control sites exhibited considerable variability. This may be caused by differences in sensor placement and the varying thicknesses of the abdominal skin regions being analyzed. To mitigate this discrepancy, multiple measurement areas should have been designated to ensure consistency in the skin thickness. Additionally, in the neck flap group, incorporating intraoperative transcutaneous oxygen monitoring before and during induced ischemia would have established a baseline measurement for the oxygen parameter, allowing for a clearer differentiation between normal and abnormal flap pO2 levels. A partial Bayesian linear mixed effects analysis revealed a statistically significant procedure-dependent change in pO2 over time (*p* = 0.002), suggesting potential differences in the healing processes between autologous transplants and neck flaps. Specifically, autologous transplants displayed more consistent, and on average lower, oxygen levels throughout the postoperative period. This may be attributed to the healing process of microvascular anastomosis, which can take up to 20 days (44). Consequently, during this time, the flap's blood supply remains dependent on a healing anastomosis that may be susceptible to leakage, constriction under tension, or the development of strictures (45). Although these conditions were not investigated in this study, they can potentially compromise flap perfusion and, subsequently, pO2 levels.

Significant limitations of this study include the small sample size (*n* = 4) and brief 20 min ischemia period, which is markedly shorter than the warm ischemia durations of 50–90 min observed in both autologous and allogeneic procedures (15). These constraints could have undermined our ability to effectively compare the gross and histological findings between the neck flap and autologous transplant groups. Additionally, measuring transcutaneous pO2 and temperature in random areas of the flap and abdominal control skin introduced variability that could have been mitigated by assessing multiple designated sites (e.g., central, proximal, and distal flap regions). As this is the first study to employ our oxygen and temperature sensor in swine, potential issues related to the thickness of animals' skin may have impacted sensor accuracy, which was not fully accounted for. Also, this study did not evaluate the autologous transplant animals for neuromuscular injury following surgery. Given that muscle constitutes a significant portion of the VRAM flap and is particularly vulnerable to ischemic damage (46, 47), future research should assess neuromuscular injury or implement a muscle-sparing approach to mitigate donor site morbidity. Furthermore, both the neck and VRAM flaps lack mucosal tissue, which limits their applicability in facial VCA research. However, the VRAM flap does contain mammary glands, which exhibit overlapping gene expression and immune functions with salivary glands (48, 49). The unique composition of this graft positions it as a potential model for allotransplantation studies exploring secretory gland rejection or dysfunction. Lastly, the use of a Penrose drain exclusively in the autologous transplants may have influenced the observed lack of postoperative complications by effectively removing excess exudate, thereby improving the healing process and reducing inflammation. This could have also affected the transcutaneous variables recorded. Addressing these limitations in future research is essential for improving the validity and applicability of our findings.

## Conclusion

We have established a porcine autologous VRAM flap model based on the external iliac vessel system. Compared to the neck flap isolation with induced ischemia procedure, this model effectively replicates the postoperative inflammatory response observed in allogenic transplants. Moreover, the survival of both autografts, along with the absence of donor site morbidity, underscores the potential safety and reproducibility of this technique. However, to validate our findings, further studies with larger sample sizes are necessary. Overall, this study highlights the critical role of control models in understanding physiological responses to surgical interventions and emphasizes their importance in achieving accurate and generalizable results in preclinical VCA research.

## Data Availability

The datasets presented in this study can be found in online repositories. The names of the repository/repositories and accession number(s) can be found here: The Harvard Dataverse, https://doi.org/10.7910/DVN/MJVPDU.
